# Hip fracture patients admitted to hospital on weekends are not at increased risk of 30-day mortality as compared with weekdays

**DOI:** 10.1186/s10195-020-00558-4

**Published:** 2020-12-02

**Authors:** Calver Pang, A. Aqil, A. Mannan, G. Thomas, F. S. Hossain

**Affiliations:** 1 Division of Surgery and Interventional Science, Royal Free Hospital, University College London, 9th Floor, 10 Pond Street, London, NW3 2PS UK; 2 Yorkshire and Humber Deanery, Yorkshire, UK; 3 Harrogate and District NHS Foundation Trust, Lancaster Park Road, Harrogate, HG2 7SX UK; 4 Airedale NHS Foundation Trust, Skipton Road, Steeton, Keighley, BD20 6TD UK; 5 Manor Hospital, Walsall Healthcare NHS Trust, Moat Road, Walsall, WS2 9PS UK

**Keywords:** Hip fracture, Hip surgery, 30-Day mortality, Weekday admission, Weekend admission, Healthcare quality

## Abstract

**Background:**

Hip fractures remain a major health concern owing to the increasing elderly population and their association with significant morbidity and mortality. The effects of weekend admission on mortality have been studied since the late 1970s. Despite most studies showing that mortality rates are higher for patients admitted on a weekend, the characteristics of the admitted patients have remained unclear. We aim to investigate this ‘weekend effect’ at our hospital in patients presenting with a hip fracture.

**Methods:**

Patients undergoing acute hip fracture surgery were identified from the local National Hip Fracture Database. Patient demographics, fracture type, co-morbidities and admission blood parameters were examined. The outcome analysed was 30-day mortality. The data were analysed with regard to day of admission, i.e. weekday (Monday to Friday) or weekend (Saturday and Sunday).

**Results:**

A total of 894 patients were included. Results demonstrated that 30-day mortality was similar on the weekend compared with the weekday (6.96% versus 10.39%, OR 0.65, 95% CI 0.36–1.14, *p* = 0.128) for patients who sustained an acute hip fracture. The total number of deaths within 30 days was 85 (69 weekday versus 16 weekend). This remained non-significant after adjusting for several variables: age and sex only (OR = 0.65, 95% CI 0.37–1.16, *p* = 0.146), age, sex, and care variables (OR = 0.59, 95% CI 0.33–1.06, *p* = 0.080), age, sex, and blood test results (OR = 0.62, 95% CI 0.35–1.12, *p* = 0.111), and all covariates (OR = 0.69, 95% CI 0.29–1.62, *p* = 0.392). In the fully adjusted model, the following variables were independent predictors of mortality: sex (male) (OR = 1.93, 95% CI 1.11–3.35, *p* = 0.019) and ASA > 2 (OR = 2.6, 95% CI 1.11–6.11, *p* = 0.028) and age (1.08, 95% CI 1.04–1.13, *p* < 0.001).

**Conclusion:**

The evidence for a ‘weekend effect’ in patients with a hip fracture is absent in this study. However, we have shown other factors that are associated with increased mortality such as increased age, male sex and higher ASA grade.

**Level of evidence:**

Level 3.

## Introduction

Hip fractures remain a major health concern owing to the increasing elderly population and association with significant morbidity and mortality. The risk of sustaining a hip fracture for women and men over the age of 50 during their lifetime is almost 11% and 3%, respectively [1]. Reports have shown that mortality following a hip fracture is high, being 30% at 1 year and 80% at 8 years [2–4], with nearly 40% of patients unable to return to their initial baseline ambulatory status [5]. It has been shown that patients who are readmitted to hospital within 30 days of discharge following a hip fracture have worse prognosis with double the risk of mortality within the first year [6, 7].

There have been recent concerns that patients who are admitted to hospital over the weekend have a higher risk of mortality compared with weekday admissions. Freemantle et al. [8] report a ‘weekend effect’ with higher relative risk of 30-day mortality on Saturday (10%) and Sunday (15%) amongst medical and surgical patients. A clear association between weekend admission and worse patient outcomes was also demonstrated in that study [8]. A study by Walker et al. [9] however, showed that biochemical and haematological blood results explained 33% of the excess mortality on Saturday and 52% on Sunday, thereby reducing the size of the ‘weekend effect’ [9]. This explained the ‘weekend effect’ as being due to the higher proportion of admissions that were categorised as high risk and therefore having a higher average risk of mortality compared with a weekday cohort of patients. For many surgical and medical conditions, this poses a source of bias in the analysis and interpretation of the ‘weekend effect’, in that more seriously ill patients may present on the weekend, while others may be able to delay presentation to a health care institution until the working week. Fragility hip fracture patients are less likely to be subjected to such initial presentation bias owing to the nature and severity of their injury. As such they form an ideal cohort to assess a true ‘weekend effect’.

Hence this study aims to compare 30-day mortality rates between weekday and weekend admissions in patients with fragility hip fractures at a district general hospital. We hypothesise that 30-day mortality rates are similar on the weekend compared with the weekday.

## Methods

### Dataset

Hip fracture patient parameters are entered prospectively into the National Hip Fracture Database (NHFD) by specially trained personnel at the trust. We used the local NHFD to identify patients with a hip fracture admitted to our trust between 2012 and 2015 following trust approval. This data was cross-referenced from the hospital’s electronic patient record to ensure accuracy. We included all patients who were registered on this database and excluded those whose records were incomplete. Collected patient variables included day of admission grouped into weekdays (Monday–Friday) and weekends (Saturday and Sunday). For the purposes of analysis, all bank holidays were classed as weekends because staffing levels throughout the hospital are similar to those of a weekend. Other data collected included type of fracture, operative details (type of surgery, date and day of surgery and time to surgery), patient demographics (age, gender, admission source, discharge destination, length of stay, abbreviated mental test (AMT) score and mobility ability), time seen by geriatrician, blood tests (pre-operative haemoglobin level, platelet and white cell count, sodium, potassium, urea, creatinine, international normalised ratio (INR), activated partial thromboplastin time (APTT) and post-operative haemoglobin and sodium), mortality (30 days), American Society of Anesthesiologists (ASA) grade, medical co-morbidity and incidence of chest or urinary tract infections during admission episode. All patient co-morbidities were collected from hospital databases as International Classification of Disease, 10th revision (ICD-10) codes and were used to calculate the Charlson co-morbidity index. Patients with incomplete datasets were excluded, as were those who were managed non-operatively. All research was performed in accordance with guidelines and regulations and approved by the Airedale NHS Foundation Trust committee.

Type of fracture was defined as either intracapsular or extracapsular according to plain radiographs. Type of surgery was categorised as either total hip replacement, hemiarthroplasty (unipolar or bipolar), dynamic hip screw (sliding hip screw), intramedullary nail or no operation (i.e. managed conservatively). Time to surgery was calculated from time of admission to accident and emergency (A&E) to operating theatre. Mobility was categorised into indoor and outdoor ability and defined as independent, single stick/crutch, two sticks/crutch/frame or wheelchair/buggy in accordance with the classifications operationalised by the National Hip Fracture Database (NHFD). ASA grade is used to establish a person’s physiological capacity to be given an anaesthetic for a procedure. The abbreviated mental test (AMT) assessment is a ten-point scoring system to assess cognitive function, which was performed pre-operatively. The AMT score was dichotomised as a score either below or above 7. This value was chosen because an AMT score of 7 or less suggests cognitive impairment [10].

### Statistical analyses

Our statistical analyses use linear and logistic regression according to whether the response variable was continuous or binary. We commenced with models with a single binary weekend (yes/no) covariate to estimate the crude (unadjusted) risk of 30-day mortality for weekday versus weekend admissions. We then added additional covariates (sex, age, AMT, ASA, neurological disorders, alcohol intake status (EtOH), chest infection, urinary tract infection (UTI), indoor and outdoor walking status, time to surgery, time to orthogeriatrician review, blood parameters including pre-operative haemoglobin concentration, white cell count, platelets counts, plasma sodium, potassium, urea, creatinine concentrations and clotting parameters of INR and APTT) on the basis of their clinical justification (not statistical criteria) to produce adjusted estimates of the ‘weekend effect’. For the logistic regression models, we report the effect sizes in terms of odds ratios (OR). Statistical significance was set at *p* < 0.05. Our results focus on size of the 'weekend effect' reported from model coefficients with 95% confidence intervals (95% CI). All analyses were carried out using Statistics and Data (STATA) software [11].

## Results

This was a retrospective study where 1085 patients with neck of femur fractures were identified but only 894 patients were included in the analysis. This was due to incomplete medical records and data missing completely at random. Table 1 presents the prevalence of patient characteristics for weekday and weekend admission. These results demonstrated that 30-day mortality was similar in the weekend cohort compared with the weekday cohort (6.96% versus 10.39%, OR 0.65, 95% CI 0.36–1.14, *p* = 0.128), with no statistical significance.

**Table 1:** Characteristics of weekend versus weekday admissions

	Weekday	Weekend	*p*-Value^†^	Weekend effect^†^ (95% CI)
Number of admissions (%)	664 (74.27)	230 (25.73)	–	–
Sex (male) (%)	184 (27.71)	59 (25.65)	0.545	Odds ratio 0.90 (0.64–1.26)
Died (30 days) (%)	69 (10.39)	16 (6.96)	0.128	Odds ratio 0.65 (0.36 to 1.14)
AMT > 7 (%)	226 (34.04)	82 (35.65)	0.657	Odds ratio 1.07 (0.78 to 1.47)
ASA > 2 (%)	464 (69.88)	165 (71.74)	0.595	Odds ratio 1.09 (0.78 to 1.52)
Neurological disorders (%)	91 (13.70)	30 (13.04)	0.801	Odds ratio 0.94 (0.60 to 1.47)
EtOH (%)	17 (2.56)	5 (2.17)	0.745	Odds ratio 0.84 (0.31 to 2.32)
Chest infection (%)	114 (17.17)	34 (17.78)	0.402	Odds ratio 0.84 (0.55–1.27)
UTI (%)	102 (15.36)	50 (21.74)	0.027	Odds ratio 1.53 (1.05 to 2.23)
Indoor walking (independent) (%)	298 (44.88)	98 (42.61)	0.550	Odds ratio 0.91 (0.67 to 1.23)
Outdoor walking (independent) (%)	270 (40.66)	90 (39.13)	0.683	Odds ratio 0.94 (0.69 to 1.27)
Mean age (SD)	82.53 (9.09)	83.09 (8.26)	0.407	0.56 (−0.77 to 1.90)
Mean time to surgery (h) (SD)	31.36 (27.50)	28.06 (30.61)	0.129	−3.30 (−7.55 to 0.95)
Mean time to geriatrician (h) (SD)	49.59 (107.27)	64.10 (206.97)	0.175	14.51 (−6.49 to 35.50)
Mean haemoglobin (g/L) (SD)	122.65 (18.96)	124.65 (18.06)	0.163	2.00 (−0.81 to 4.82)
Mean white cell count (10^9^/L) (SD)	10.41 (4.84)	11.76 (12.71)	0.021	1.35 (0.20 to 2.51)
Mean platelets (10^9^/L)(SD)	251.28 (89.47)	238.68 (75.55)	0.056	−12.60 (−25.23 to 0.33)
Mean sodium (mmol/L) (SD)	136.57 (6.48)	137.37 (12.05)	0.210	0.79 (−0.45 to 2.04)
Mean potassium (mmol/L) (SD)	4.28 (0.66)	4.25 (0.85)	0.542	−0.03 (−0.14 to 0.07)
Mean urea (mmol/L) (SD)	8.05 (4.25)	8.50 (4.89)	0.195	0.44 (−0.22 to 1.10)
Mean creatinine (μmol/L) (SD)	87.22 (46.00)	93.28 (56.54)	0.106	6.06 (−1.28 to 13.41)
Mean INR (SD)	1.06 (0.13)	1.05 (0.13)	0.451	−0.01 (−0.03 to 0.01)
Mean APTT (s) (SD)	28.44 (4.09)	29.83 (18.15)	0.066	1.38 (−0.09 to 2.87)

^†^*p*-Values and estimates (95% CI) of effect are from a weekend term in a linear (for continuous variables) or logistic (for binary variables) regression model

There was no difference in AMT score status (cutoff being 7) [10] between the two groups (34.04 versus 35.65%, OR 1.07, 95% CI 0.78–1.47, *p* = 0.657). Likewise, ASA grade > 2 showed similar mortality rates between the two groups (69.88% versus 71.74%, OR 1.09, 95% CI 0.78–1.52, *p* = 0.595).

Average time to surgery (30.61 versus 27.50 h, 95% CI −7.55 to 0.95, *p* = 0.129) and time to geriatrician (64.10 versus 49.59 h, 95% CI −6.9 to 35.50, *p* = 0.175) were longer at weekends, but both groups met the 36-h target for surgery and 72-h target for geriatric assessment.

Urinary tract infection during admission showed a higher prevalence on the weekend compared with the weekday (21.74% versus 15.36%, OR 1.53, 95% CI 1.05–2.23, *p* = 0.027). Likewise, higher mean white cell count pre-operatively showed higher prevalence on weekend compared with weekday (11.76 versus 10.41, 95% CI 0.20–2.51, *p* = 0.021).

**Fig. 1:**
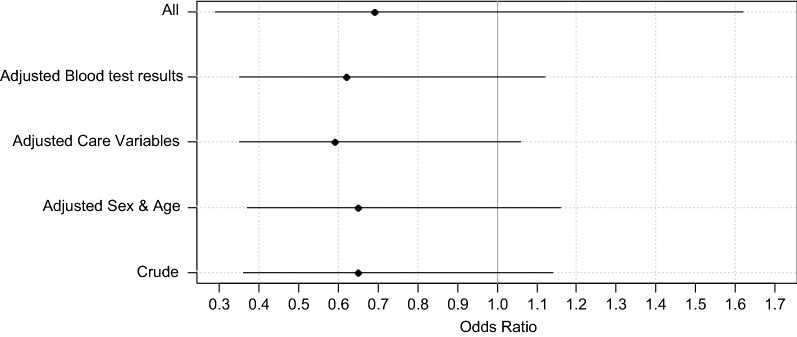
Five models reported odds ratios (with 95% confidence intervals) for death in hospital within 30 days, following emergency admissions over the weekend versus weekdays

The crude (unadjusted) 30-day mortality rate for hip fracture patients following weekend admissions (Table 2; Fig. 1) was similar to the weekday admissions (OR = 0.65, 95% CI 0.36–1.14, *p* = 0.128). This remained non-significant after stepwise adjustments for several variables: age and sex only (OR = 0.65, 95% CI 0.37–1.16, *p* = 0.146), age, sex, and care variables (OR = 0.59, 95% CI 0.33–1.06, *p* = 0.080), age, sex, and biochemical parameters (OR = 0.62, 95% CI 0.35–1.12, *p* = 0.111), and all covariates (OR = 0.69, 95% CI 0.29–1.62, *p* = 0.392).

**Table 2:** Odds ratios (95% CI, *p* values) for 30-day mortality comparing weekend versus weekday admissions

Weekend effect	Odds ratio (95% CI)
Crude (unadjusted) odds ratio for death following weekend admission	0.65 (0.36–1.14) *p* = 0.128
Odds ratio for death following weekend admission adjusted for sex and age	0.65 (0.37–1.16) *p* = 0.146
Odds ratio for death following weekend admission adjusted for age, sex, AMTS, ASA, time to surgery and time to geriatrician	0.59 (0.33–1.06) *p* = 0.08
Odds ratio for death following weekend admission adjusted for age, sex and all blood test results (haemoglobin, white cell count, platelets, sodium, potassium, urea, creatinine, INR, APTT)	0.62 (0.35–1.12) *p* = 0.111
Odds ratio for death following weekend admission adjusted for AMTS, ASA, time to surgery, time to geriatrician and all blood test results	0.69 (0.29–1.62) *p* = 0.392

In the fully adjusted model, the following variables were independent predictors of mortality: sex (male) (OR = 1.93, 95% CI 1.11–3.35, *p* = 0.019), ASA > 2 (OR = 2.6, 95% CI 1.11–6.11, *p* = 0.028) and age (1.08, 95% CI 1.04–1.13, *p* < 0.001).

## Discussion

### Statement of principal findings

In this study, we found that mortality following hip fracture is no greater for patients who are admitted on a weekend compared with weekday admissions. The crude estimate of the odds ratio changed very little as a result of the adjusted models: crude model (OR = 0.65, 95% CI 0.36–1.14, *p* = 0.128) and fully adjusted model (OR = 0.69, 95% CI 0.29–1.62, *p* = 0.392).

### Strengths and weaknesses of the study

Our study was conducted in a district general hospital within a trauma and orthopaedic department where care is co-ordinated by a dedicated trauma nurse practitioner, foundation doctors, core surgical trainees, orthopaedic registrars and nominated consultant. The department runs a 7-day consultant-led service where all neck of femur patients are seen on the morning ward round, and hip fracture operating lists run every day of the week. It has been shown that treatment of hip fractures on dedicated trauma lists reduces morbidity and mortality [12]. In addition, operative delays of more than 48 h have been shown to increase risk mortality. Therefore, in hospital settings where there is no 7-day service of available operating theatre and surgeons, this could potentially influence the mortality rates [13, 14]. All data collected were from the NHFD and cross-referenced against the hospital’s electronic database to ensure accuracy. Additionally, we were able to collect covariate data over and above what is available in the NHFD database, which could influence the mortality rate.

Our study does have limitations. As shown from our results, we lost 190 patients from our initial sample size. Despite all efforts in trying to collate all information from both the trust and NHFD database, these are subject to error during data entry, which ultimately affects the accuracy of our analysis. However, in our study, the point estimate for the ‘weekend’ effect was in the opposite direction to that supposed and remained consistently so following adjustment. In addition, we did not conduct a power analysis to determine sample size, therefore we did not know the number of subjects required to generate the effect.

### Strengths and weaknesses in relation to other studies

Many studies have reported data on the ‘weekend effect’, but not many have looked into other potential prognostic factors. Thomas et al. [15] reported a significantly increased association between weekend admission and 30-day mortality for patients undergoing hip fracture surgery, but there was no increase in mortality associated with weekend surgery. In comparison with our study, the authors did not adjust for confounding factors of pre-morbid function and cognitive impairment, which have been shown to be important prognostic information on mortality [16]. Matthews et al. [17] showed no statistical difference in either 30- or 120-day mortality between hip fracture patients admitted on weekdays or weekends. This study addressed effects on both 30-day and 120-day mortality in a sample size of 816 patients, which is comparable to our study, with similar results. Our findings are further supported by a significantly larger nationwide study of over 340,000 hip fracture patients from the USA [18], which showed that patients admitted on weekends had lower mortality compared with weekday admission and did not support a ‘weekend effect’. However, the study was conducted in the USA, so these patients may have been subject to different management and staffing protocols. Sayers et al. [19] however, in a national cohort of over 240,000 patients from the NHFD, showed that day of admission was not associated with 30-day mortality. However, the study demonstrated that surgery on a Sunday and delay to surgery of more than 24 h were both associated with a 9.4% increase in 30-day mortality. In addition, discharge on a Sunday or out of hours was associated with a 51.5% and 17.4% increase in 30-day mortality, respectively. Although our study did not report mortality rate by days of the week and association of mortality with day of discharge, our findings of an absence of a ‘weekend effect’ in hip fracture patients is in agreement with that of Nandra et al. [20], which showed a reduced mortality with weekend admissions compared with weekday. This study also reported a higher mortality risk in patients with cognitive decline and pre-existing co-morbidities.

Our results further confirmed that patients who were older, male or had a higher ASA grade had a higher mortality risk, which is in concordance with previous studies pertaining to early mortality after hip fracture treatment [21–24].

Delay in time to surgery has consistently been demonstrated to be an important risk factor for mortality amongst fragility hip fracture patients [20, 25] and forms an integral part of the NICE guidelines on hip fracture management. Existing literature has shown that certain diagnoses are associated with increased mortality with weekend admissions due to fewer available staff and more restricted access to clinical services. Freemantle et al. [8] showed an increased 30-day mortality risk for patients admitted with emergency conditions over the weekend period compared with weekdays. Studies outside of UK have shown that ruptured aortic aneurysm [26], pulmonary embolism [26] and duodenal ulcers [27] have a significantly increased risk of mortality if admitted on a weekend compared with a weekday. This suggests that the ‘weekend effect’ may be more applicable to presenting complaints that require time-critical diagnosis and imminent treatment. However, neck of femur fractures are not time critical, and therefore our study findings may not be unexpected. Indeed, they are consistent with other published studies.

### Meaning of the study: possible mechanisms and implications for clinicians or policymakers

This study has demonstrated no evidence of a ‘weekend effect’ amongst patients with hip fractures, with a small reduction in 30-day mortality with weekend admissions compared with weekday admissions. This data is consistent with many reports that have studied mortality rate amongst patients with hip fractures. The National Institute for Health and Clinical Excellence (NICE) have published guidelines on managing fragility hip fractures in adults. These recommendations are based on surgical, anaesthetic and orthogeriatric evidence and expertise. The key elements of adherence continue to include early surgery and co-ordinated care through a multidisciplinary approach to help faster recovery and restoration of mobility.

### Future work

A study with a larger sample size is required to reduce any uncertainty. In addition, it would be useful to compare results amongst large teaching hospitals and district general hospitals to assess whether there is any statistical difference with staffing and resources.

## Conclusion

The evidence for a ‘weekend effect’ in patients with a hip fracture is absent in this study. However, we have shown other factors that are associated with an increased mortality such as increased age, male sex and higher ASA grade.

## Data Availability

The datasets used and/or analysed during the current study are available from the corresponding author on reasonable request.
